# Controlling therapeutic protein expression via inhalation of a butter flavor molecule

**DOI:** 10.1093/nar/gkac1256

**Published:** 2023-01-10

**Authors:** Adrian Bertschi, Bozhidar-Adrian Stefanov, Shuai Xue, Ghislaine Charpin-El Hamri, Ana Palma Teixeira, Martin Fussenegger

**Affiliations:** Department of Biosystems Science and Engineering, ETH Zurich, Mattenstrasse 26, CH-4058 Basel, Switzerland; Department of Biosystems Science and Engineering, ETH Zurich, Mattenstrasse 26, CH-4058 Basel, Switzerland; Department of Biosystems Science and Engineering, ETH Zurich, Mattenstrasse 26, CH-4058 Basel, Switzerland; Département Génie Biologique, Institut Universitaire de Technologie, Université Claude Bernard, Lyon 1 Villeurbanne Cedex F-69622, France; Department of Biosystems Science and Engineering, ETH Zurich, Mattenstrasse 26, CH-4058 Basel, Switzerland; Department of Biosystems Science and Engineering, ETH Zurich, Mattenstrasse 26, CH-4058 Basel, Switzerland; University of Basel, Faculty of Science, Mattenstrasse 26, CH-4058 Basel, Switzerland

## Abstract

Precise control of the delivery of therapeutic proteins is critical for gene- and cell-based therapies, and expression should only be switched on in the presence of a specific trigger signal of appropriate magnitude. Focusing on the advantages of delivering the trigger by inhalation, we have developed a mammalian synthetic gene switch that enables regulation of transgene expression by exposure to the semi-volatile small molecule acetoin, a widely used, FDA-approved food flavor additive. The gene switch capitalizes on the bacterial regulatory protein AcoR fused to a mammalian transactivation domain, which binds to promoter regions with specific DNA sequences in the presence of acetoin and dose-dependently activates expression of downstream transgenes. Wild-type mice implanted with alginate-encapsulated cells transgenic for the acetoin gene switch showed a dose-dependent increase in blood levels of reporter protein in response to either administration of acetoin solution via oral gavage or longer exposure to acetoin aerosol generated by a commercial portable inhaler. Intake of typical acetoin-containing foods, such as butter, lychees and cheese, did not activate transgene expression. As a proof of concept, we show that blood glucose levels were normalized by acetoin aerosol inhalation in type-I diabetic mice implanted with acetoin-responsive insulin-producing cells. Delivery of trigger molecules using portable inhalers may facilitate regular administration of therapeutic proteins via next-generation cell-based therapies to treat chronic diseases for which frequent dosing is required.

## INTRODUCTION

Trigger-inducible gene switches are important tools in biology research and for synthetic biology applications, including gene and cell-based therapies. In recent years, the range of synthetic gene regulatory systems responsive to small molecules (e.g. caffeine (1), spearmint (2), menthol (3)) or physical stimuli (e.g. heating (4)), light (5) or electrical signals (6)) has increased considerably, offering multiple options for specific in vivo gene regulation. To achieve clinical relevance, such systems should be strictly controllable by nontoxic and inexpensive inducers that can be easily administered in order to encourage patient compliance. Furthermore, in the case of food components, the range of activation concentrations should be higher than the endogenous levels following food intake or inadvertent external stimulation. Small molecule triggers have been delivered by oral ingestion (7,8), by transdermal application (9), and by inhalation (2). Among them, inhalation is a particularly attractive strategy for systemic administration of (semi-)volatile molecules, enabling rapid absorption owing to the large surface area of the pulmonary alveoli connected to a vast network of blood capillaries. A further advantage is the avoidance of potential degradation by trigger-metabolizing gut bacteria, in contrast to orally administered molecules. However, no synthetic gene regulatory system that can be activated by inhalation has yet met all the criteria for practical application. For instance, the spearmint inducible system (2) can be unintentionally activated by exposures occurring during everyday life.

In this work, we focused on the volatile small molecule acetoin to engineer a mammalian gene switch that can be activated in vivo by inhalation. Acetoin is a non-toxic food additive. It is actively metabolized in mammals to the secondary alcohol 2,3-butanediol, which in turn is reduced to 2-butanone or 2-butanol and eventually cleared from the body with a half-life of about 0.9 h (10). When humans are exposed to large quantities of ethanol, a slight increase of blood acetoin can be detected (11), but as the production of acetoin is slower than its reduction, there is no accumulation of acetoin. Using a small trigger molecule that is actively degraded would enable better control of dosing and therefore better regulation of the activity of the target gene circuit.

Several bacteria, mostly from the *Enterobacteriaceae* family, produce acetoin as by-product when growing in glucose media, and use acetoin as an energy source when glucose is depleted (12). Acetoin binds to the regulatory protein AcoR, leading to induction of acetoin-metabolizing enzymes (13). Structurally, AcoR contains a helix-turn-helix DNA binding domain, an ATP-binding domain, an acetoin binding pocket and a sigma54 recruiting domain (14). The natural operator sequence to which the helix-turn-helix domain binds is located approximately 80 to 100 bp upstream of the start codon at a palindromic region of around 30 bp (15,16), but the nature of the interaction of AcoR with the DNA has not been fully characterized. In the presence of acetoin, AcoR shows increased binding affinity to the DNA and initiates transcription of the regulated genes.

In order to develop a synthetic gene circuit to control therapeutic gene expression using a commercial portable inhaler, we generated a stable HEK293T cell line that constitutively expresses AcoR from *Bacillus subtilis* fused to a mammalian transactivator (P_mPGK_-VP16_f-type_-AcoR-pA_bGH_) and a transgene under the control of the operon of the acetoin-responsive AcoR (P_OAcoR_-P_hCMVmin_-SEAP-pA_bGH_). AcoR is only activated by high concentrations of acetoin, and is not activated even by natural products containing substantial amounts of acetoin. To test the potential utility of this system, we first induced the engineered cells in vitro either by direct exposure to acetoin in the medium or by exposure to acetoin vapor. Then, as a proof of concept, we translated the findings to an in vivo mouse model, in which gene expression was induced by exposure to acetoin aerosol generated with a commercial portable inhaler.

## MATERIALS AND METHODS

### Plasmid design and molecular cloning

All plasmids used and constructed in this study are listed in Supplementary Table 1. Plasmids were designed using Benchling (www.benchling.com) and sequences were verified at Microsynth AG, Switzerland.

The bacterial strain XL10 gold K12 *E. coli* (Stratagene) was used to propagate the plasmids. Bacteria were grown at 37°C in Lurie-Bertani lysogeny broth under shaker-aeration. Plasmid DNA was extracted after an 8–16 h incubation period using a Zippy Plasmid Mini-Prep Kit (Cat. No. D4037, Zymo Research) according to the manufacturer's instructions. For PCR reactions Phusion High-Fidelity DNA polymerase (Cat. No. F530, ThermoFisher Scientific, Rheinach, Switzerland) was used according to the manufacturer's instructions. For phosphorylation and annealing of two oligonucleotides, T4 Polynucleotide Kinase (Cat. No. M0201, New England BioLabs) was used in T4 DNA ligase buffer (Cat. No. B69, ThermoFisher Scientific) according to the manufacturer's instructions.

Plasmid DNA or purified PCR-amplified sequences (300–1000 ng) were digested using 2 units of standard restriction enzyme (New England BioLabs) and calf intestinal alkaline phosphatase (Quick CIP, Cat. No. M0525, New England BioLabs) for an incubation period of 2 h at the manufacturer's recommended temperature. The digestion products were purified by standard agarose gel electrophoresis. DNA fragments were extracted using the Zymoclean Gel DNA Recovery Kit (Cat. No. D4002, Zymo Research) according to the manufacturer's instructions. Purified DNA fragments were then ligated to the counterpart with the matching overhang using T4 DNA ligase (Cat. No. EL0011, ThermoFisher Scientific) in T4 DNA ligase buffer (Cat. No. B69, ThermoFisher Scientific) for at least 20 min before transformation into competent bacteria. To do so, 20–50 μl of competent bacteria was added to 10 μl of the ligation mix and heat-shocked at 42°C for 45 s. Heat-shocked bacteria were plated onto ampicillin-containing LB-agar and incubated for 16 h, then a colony was picked and grown as previously described.

***Chemicals*.** Ethanol (EtOH; Cat. no. 51976), dimethyl sulfoxide (DMSO; Cat. no. D4540), acetoin (Cat. no. W200808), lactic acid (Cat. no. L7022), pyruvate (Cat. no. P3662), 2,3-butanedione (Cat. no. B0753), and 4-hydroxy-3-hexanone (Cat. no. CDS000463) were purchased from Sigma-Aldrich (Buchs, Switzerland).

***Cell culture and transfection*.** Human embryonic kidney cells (HEK-293T, ATCC: CRL-11268), HeLa cells (HeLa, ATCC: CCL-2), bone marrow-derived immortalized mesenchymal stem/stromal cells (hMSC-TERT) cells (17), Hep G2 cells (HEPG2, ATCC: HB-8065), baby hamster kidney cells (BHK, ATCC: CCL-10) and HT-1080 cells (HT1080, ATCC: CCL-121) were cultivated in Dulbecco's modified Eagle's medium (Gibco™ DMEM, Cat. no. 31966–021, ThermoFisher Scientific), with 1% (v/v) streptomycin/penicillin (Gibco™ Penicillin-Streptomycin, Cat. no. 15070–063, ThermoFisher Scientific) and supplemented with 10% (v/v) fetal bovine serum (FBS, Cat. no. F7524, Sigma-Aldrich). Chinese hamster ovary cells (CHO-K1, ATCC: CCL-61) and A549 cells (A549, ATCC: CCL-185) were cultivated in Ham's F-12K (Kaighn's) medium (Gibco™ F-12K, Cat. no. 21127–022, ThermoFisher Scientific) with 1% (v/v) streptomycin/penicillin (Gibco™ Penicillin-Streptomycin, Cat. no. 15070-063, ThermoFisher Scientific) and supplemented with 10% (v/v) fetal bovine serum (FBS, Cat. no. F7524, Sigma-Aldrich). For optimal consistency between different transient transfections, the protocol was standardized. 15 000 cells were transfected overnight with a total of 150 ng of DNA per well in 96-well plates, using a ratio of DNA to polyethyleneimine (PEI, Cat. No. 24765-2, Polysciences) of 1:6. The PEI (MW 40 000) stock solution was 1 mg/mL in ddH_2_O. The next morning the medium was exchanged for inducer-containing medium and after 24 h induction (unless otherwise stated) the culture supernatant was collected for quantification of secreted reporter protein.

***SEAP reporter assay*.** SEAP reporter assay was performed as previously described (18). Briefly, culture supernatant containing SEAP was heat-inactivated at 65°C for 30 min. Then, 20 μl of inactivated medium was diluted with 80 μl of water. Next, 80 μl of 2× SEAP assay buffer (20 mM homoarginine, 1 mM MgCl_2_, 21%(v/v) diethanolamine, pH 9.8) and 20 μl of substrate solution (120 mM *p*-nitrophenyl phosphate in 2× SEAP assay buffer (Acros Organics BVBA)) was added and the time-dependent increase in absorbance at 405 nm was measured using a Tecan Infinite M1000 microplate reader over a period of 30 min. The presented values are representative measurements of three independent experiments.

***Western-blot analysis*.** HEK293T cells were seeded and transfected with FLAG-tagged AcoR-VP16_f-type_ from different *Bacillales* under the control of the constitutive P_PKG_ promoter. At 48 h after transfection, cells were harvested, centrifuged at 1000 × g for 2 min, washed twice with ice-cold PBS and lysed using RIPA buffer supplemented with a protease inhibitor cocktail (Pierce™ Protease Inhibitor Tablets, Cat. no. A32963, ThermoFisher Scientific) on ice for 20 min with repeated vortexing. The lysate was then centrifuged at 12 000 × g for 30 min at 4°C and the clear lysate was transferred into a new tube. Protein concentration was determined using a quantification kit (Pierce™ BCA Protein Assay Kit, Cat. no. 23227, ThermoFisher Scientific). 5X reduced Laemmli sample buffer was added and the mixture was heated for 5 min at 95°C. An aliquot containing 10 μg of protein was loaded on SDS-PAGE. After SDS-PAGE, the gel was blocked using 10% skimmed milk in TBST buffer at room temperature for 1 h. FLAG-specific primary antibody (monoclonal ANTI-FLAG® M2 antibody produced in mouse, Cat. no. F1804, Sigma-Aldrich) and β-actin specific primary antibody (β-Actin (13E5) Rabbit mAb, Cat. no. 4970, Cell Signaling) were used at 1:1000 dilution. Secondary HRP-conjugated goat-anti-rabbit (HRP-AffiniPure Goat Anti-Rabbit IgG (H + L), Cat. no. 111–035-144, Jackson Immunoresearch) and anti-mouse (HRP-AffiniPure Goat Anti-Mouse IgG (H + L), Cat. no. 115-035-003, Jackson Immunoresearch) antibodies were used at a dilution of 1:10 000. A protein ladder (PageRulerTM Plus Prestained Protein Ladder, 10–250 kDa, Cat. no. 26619, ThermoFisher Scientific) was used as protein molecular weight marker. An imaging platform (FUSION Pulse TS, Cat. no. 37480003, Vilber, France) was used to develop blots before they were analyzed using Adobe Illustrator.

***RNA sequencing*.** HEK293T cells were seeded and transfected as previously described. Cells were collected 24 h after induction with acetoin or control medium. RNA was then isolated using a Quick-RNA MiniPrep Kit (Cat. no. R1055, Zymo Research). The isolated RNA was prepared for sequencing with a TruSeq stranded mRNA Illumina HT kit v2 and sequenced with a NextSeq 500 using Illumina RTA v 2.11.3 with 76 cycles. Illumina sequencing data were demultiplexed and primary analysis was performed using a Snakemake workflow, which includes Trimmomatic (v 0.35), alignment to the GRCh38 genome with Hisat2 (v 2.1.0), Samtools (v 1.9) to sort and index the alignment BAM files, and Counts from the Subread package (v 2.0.1) to count reads in the gene ranges, using human Ensembl annotation v105. The count vectors for all samples were combined into a table, which was then subjected to the secondary analysis in R.

Quality control and sample consistency were confirmed with PCA, using R package PCATools. The count table was processed for secondary (statistical) analysis with R scripts using EdgeR (v3.32), affording lists of genes ranked for differential expression by *P*-value. Benjamini–Hochberg adjusted *P*-value was used to estimate the false discovery rate. Pathway enrichment analysis was performed with GeneGo Metacore. (Primary analysis workflow repository: https://github.com/michalogit/snake_hisat/)

***Stable cell line generation*.** For stable cell line production, we employed the Tier-3 vector described by Haellman et al. (19) using the Sleeping Beauty transposon protocol (20). 25 000 HEK293T cells were seeded into a well of a 6-well plate and transfected with pAB800 (2000 ng), pAB801 (400 ng) and pTS395 (400 ng) overnight. Next morning, the transfection mix was replaced by standard cell culture medium. After 24 h, antibiotic selection was started by addition of 2 μg ml^−1^ puromycin (Cat. no. A1113803; ThermoFisher Scientific) and 10 μgml^−1^ blasticidin (Cat. no. A1113903; ThermoFisher Scientific). Single cells were sorted using standard FACS according to the expression level of the integrated fluorophores. Single cells were seeded into wells of a 96-well plate containing conditioned media, where they were grown for two weeks before testing their performance. Similarly, the AIGES_Ins_ monoclonal cell line was generated by transfecting HEK-293T cells with the plasmids pAB804, pBS894 and pTS395 and selected with 100 μg ml^−1^ zeocin (Cat. no. R25005; ThermoFisher Scientific).

***Animal experiments*.** For intraperitoneal implantation into mice, the transgenic HEK cells were alginate-encapsulated using an Inotech Encapsulator Research Unit IE-50R (EncapBioSystem Inc., Greifensee, Switzerland) with a 200 μm nozzle, a vibration frequency of 1,024 Hz, 1200 V and a flow rate of 400 units using a 25 ml syringe (21). Functionality of encapsulated cells was tested in vitro and capsule homogeneity as well as integrity was confirmed by means of microscopy. 25 000–50 000 capsules per mouse containing 200 cells per capsule were implanted intraperitoneally. All experiments involving animals were performed according to the directive of the European Community Council (2010/63/EU), approved by the French Republic (project no. DR2018-40v5, APAFIS #16753), and carried out by Ghislaine Charpin-El Hamri (license no. 69266309) at the Institut Universitaire de Technologie of the Université Claude Bernard Lyon 1, F-696226, Villeurbanne Cedex, France.

***In vivo induction protocols*.** Encapsulated cells were implanted intraperitoneally into C57BL/6 mice (female, 4–6 weeks, Janvier) in 1 ml of MOPS buffer. For the experimental type-1 diabetes study, we used C57BL/6 mice (male, 4–6 weeks, Janvier) treated with 70 mg/kg per mouse per day streptozocin (STZ, Cat. No. S0130, Sigma-Aldrich) for 4 days to deplete the insulin production of beta cells (22). Cells were implanted 24 h prior to the induction for all experiments. The mice were then treated with 0–7500 mg/kg acetoin dissolved in 100 mM Tris–HCl (Tris base, Cat. No. 200923, Biosolve; HCl, Cat. No. 320331, Sigma-Aldrich) at pH 7.2 by oral gavage or given lychees, liquid butter or cheddar cheese, purchased at a local supermarket. Lychees in the form of a smoothie and butter were given by oral gavage (200 μl per mouse), while cheese was presented as a block after removal of other food sources; 18 g of cheese was consumed overnight by 6 mice. For induction of the encapsulated cells via an inhaler (EMSER, Sidro AG, Rheinfelden, Switzerland), a solution of 600 mg/ml acetoin in 100 mM Tris–HCl was vaporized into a 11.5 × 6 × 8 cm box with an induction cycle of 150 s on, 150 s off for 0–30 min. All data was collected at 12 h after the induction, unless otherwise stated.

***GTT Assay*.** Mice were fasted for 8 h before i.p. injection with a glucose solution (1.5 g/kg). After the glucose injection, blood glucose level was monitored every 15 min for 60 min and every 30 min for another 60 min using a glucometer (Contour XT, BAYER HealthCare, Leverkusen, Germany) to generate a glycemia profile over the time course of 120 min.

***Insulin ELISA*.** Ultrasensitive Mouse Insulin ELISA (Cat. No. 10-1132-01, Mercodia) was used according to the manufacturer's instructions to measure blood insulin levels. Absorbance was measured at 450 nm using a Tecan Infinite M1000 microplate reader.

***Inflammation marker assays*.** Encapsulated cells were implanted into TD1 mice, cells were induced by oral gavage application of acetoin [5 g/kg] body weight at 2 and 4 days after cell implantation and blood serum was collected at 12 h after the second acetoin application. Blood serum was then analyzed using a mouse IL-6 high-sensitivity ELISA kit (BMS603HS, Invitrogen), a mouse INF alpha high-sensitivity ELISA kit (BMS607HS, Invitrogen) and a mouse IFN gamma ELISA kit (BMS606-2, Invitrogen), according to the manufacturer's instructions.

***Statistical analysis*.** Variation was determined using standard deviation and presented as error bars within the graphs. Standard error of mean was used to present the variation of the *in vivo* data. To examine significance, statistical evaluation was conducted using the unpaired two-tailed student t-test and one-way ANOVA analysis for comparison of two datasets or multiple datasets respectively. Results are indicated in the graph as followed: *P < 0.05, **P < 0.01, ***P < 0.001, ****P < 0.0001. All statistical evaluations were conducted using the implemented algorithms of Graphpad Prism 8 (GraphPad Software Inc., San Diego, California, USA)

***Data availability*.** All plasmids and data generated in this study are available on request. Requests for materials should be made to the corresponding author.

## RESULTS

### Design and in vitro evaluation of the acetoin-inducible gene switch

**Figure 1.**
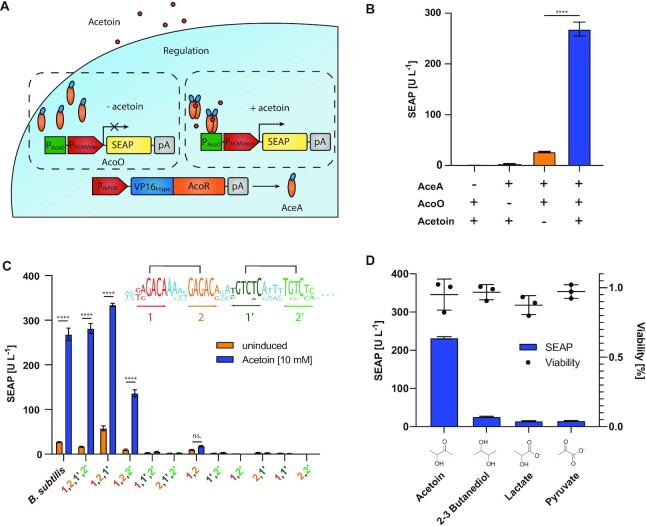
(**A**) Schematic overview of the design and function of the acetoin-inducible gene switch (AIGES). The *Bacillus subtilis*-derived acetoin transcriptional regulator AcoR was C’-terminally fused to a minimal version of the VP16 viral transactivation domain f-type (VP16_f-type_). This fusion protein (VP16_f-type_-AcoR), which we called acetoin-responsive mammalian transactivator AceA, is controlled by the constitutive human phosphoglycerate kinase promoter (P_mPGK_) (pAB003, P_mPGK_-AceA-pA). A reported AcoR binding region was cloned 5’ to the minimal human cytomegalovirus immediate early promoter (P_hCMVmin_) to control the expression of the reporter protein, secreted alkaline phosphatase (SEAP), in an acetoin-responsive manner (pAB008, O_AceA_P_hCMVmin_-SEAP-pA). AceA interacts with acetoin and binds the response element to start transcription of SEAP. (**B**) SEAP levels in the supernatants of HEK cells transfected with the AIGES plasmids (pAB003 and pAB008), cultured in medium either containing acetoin (10 mM), or in acetoin-free medium, and then left for 24 h before SEAP measurement. Due to the volatility of acetoin, induced and non-induced cells were cultured in separate plates and kept in different shelves inside the incubator. (**C**) Characterization of the binding motif of AcoR within the reported binding region. SEAP secretion from HEK cells co-transfected with pAB003 and the natural operator sequence of *Bacillus subtilis* (pAB008), the consensus sequence of five *Bacillales* (pAB101) or variants of truncations of the palindromic consensus sequence (pAB102-pAB111), which comprises two homologous pairs of binding sites, 1 and 2, and in combination with 1’ and 2’ form a palindromic repeat. (**D**) The specificity of AceA for acetoin was demonstrated by adding structurally similar molecules and measuring the reporter response. Toxicity of all tested compounds was measured using a resazurin cell viability assay. All experiments were performed in triplicates and repeated at least three times.

To engineer an acetoin-responsive mammalian transactivator (AceA), we fused the *Bacillus subtilis* AcoR protein C’-terminally to the minimal acidic activation domain of the herpes simplex virus VP16 transactivation domain (VP16_f-type_). To test the ability of AceA to regulate gene expression in mammalian cells, we designed a synthetic promoter consisting of the full 30 bp operator sequence previously reported for AcoR (16) upstream of a minimal version of the human cytomegalovirus immediate-early promoter (P_hCMVmin_) and used it to drive expression of human placental secreted alkaline phosphatase (SEAP) as a reporter protein (Figure 1A). Human embryonic kidney (HEK293-T) cells co-transfected with this reporter plasmid (P_hCMVmin_-SEAP-pA; pAB008) and a construct encoding constitutive expression of AceA driven by the mouse phosphoglycerate kinase 1 promoter (P_mPGK_-AceA; pAB003) showed induction of SEAP expression in the presence of acetoin (Figure 1B).

To characterize the binding region of AceA, we analyzed the AcoR operator site consensus sequence (pAB101) from five different Bacillales (*B. subtilis, B. licheniformis, B. pseudomonas, B. cereus* and *B. pumilus*) using Jalview (23) and replaced different regions with a confirmed non-binding nucleotide sequence of the same length. The consensus sequence reveals two regions in each strand, designated 1 and 2 or 1’ and 2’, that form a palindrome (Figure 1C). Removal of either 1 or 2 results in the loss of AceA-regulated gene expression and inducibility by acetoin. Replacing both 1’ and 2’ results in the loss of induction, but the same level of basal expression is retained. The replacement of either 1’ or 2’ does not impair the induction of gene expression by acetoin. However, the replacement of 2’ increased the basal expression by a factor of 5. Based on these results, we selected the full consensus sequence as the AceA response element to place upstream of a minimal promoter controlling reporter gene expression (O_AceA_P_hCMVmin_-SEAP-pA; pAB101).

Next, to evaluate the specificity of the AceA towards acetoin, we tested if a set of structurally similar molecules can activate reporter gene expression in HEK293T cells transfected with the acetoin-inducible gene switch (AIGES). None of the tested compounds increased SEAP production (Figure 1D). Resazurin assay showed that the tested compounds had no effect on cell viability at the concentration of 10 mM. These results indicate high selectivity of AceA for acetoin, which is desirable for precise regulation of gene expression. Furthermore, RNA sequencing only revealed a total of 156 differentially expressed genes out of over 60 000 analyzed genes with a false discovery rate <20%. Both the fold changes and expression intensities of those genes were low, suggesting that acetoin had little or no effect other cellular pathways (Supplementary Figure S1).

### Optimization of the acetoin-inducible gene switch

**Figure 2.**
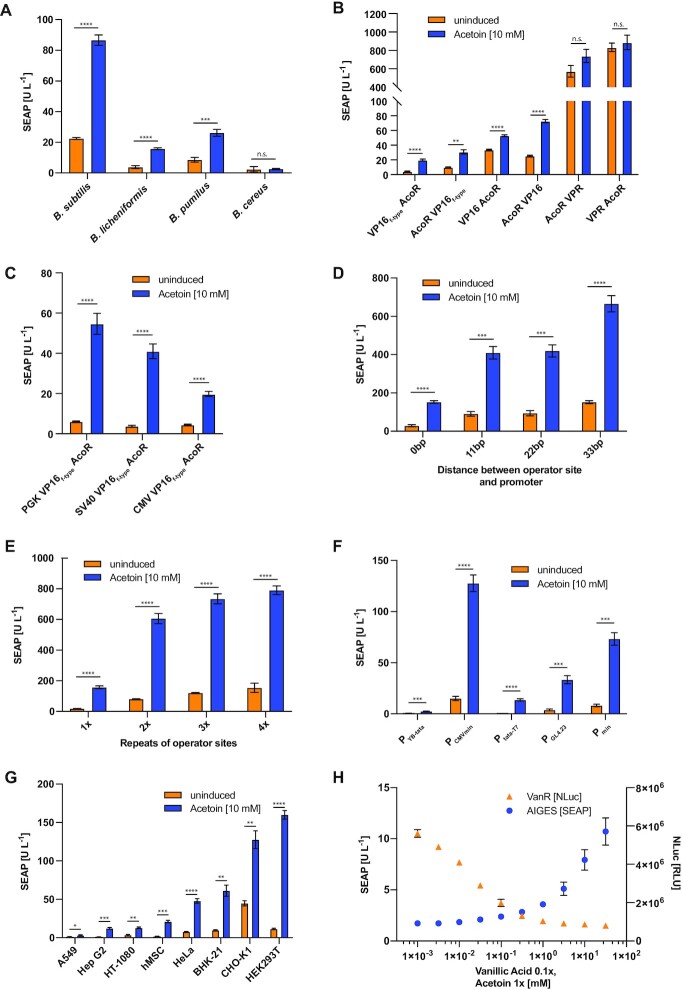
(**A**) Comparison of the ability of AcoR proteins from different *Bacillales* fused to VP16_f-type_ to activate SEAP expression in the presence of acetoin. (**B**) Effect of C- and N-terminal fusion of the viral transactivators VPR, VP16 and VP16_f-type_ on SEAP expression. (**C**) VP16_f-type_-AcoR fusion under the control of different constitutive promoters and its effect on SEAP expression. (**D**) Influence of different spacers between the operator site and the minimal promoter. (**E**) Effect of multiple operator sites on SEAP expression. (**F**) Impact of different minimal promoters on the performance of the acetoin-inducible gene switch. (**G**) Acetoin-inducible gene expression across various mammalian cell lines transfected with pAB003 and the reporter pAB101. SEAP activity was assayed after 24 h incubation of HEK and CHO-K1 cells, and after 72 h incubation for the remaining cell lines. (**H**) Orthogonality of the acetoin and vanillic acid gene switches. HEK cells were co-transfected with both the switch ON AIGES system and the switch OFF system based on vanillic acid (VA) responsive transcription factor (P_hCMV_-VanR-VP16-pA), which in the absence of VA binds to a synthetic promoter controlling NLuc expression (O_VanR_P_min_-NLuc-pA). The dose-response of SEAP and NLuc reporter proteins was evaluated in medium containing acetoin and vanillic acid. All experiments were performed in triplicate and repeated at least three times.

With the aim of further enhancing the performance of the AIGES, we next tested various modifications of AceA and the reporter construct. First, we compared the performance of the AcoR proteins from *B. subtilis, B. licheniformis, B. cereus* and *B. pumilus* fused to the VP16_f-type_ transactivation domain in HEK293T cells co-transfected with the reporter plasmid. Except for the *B. cereus* protein, all other AcoR proteins showed acetoin-responsiveness and *B. subtilis* AcoR showed the greatest induction (Figure 2A). Although HEK293T cells expressed the proteins derived from various *Bacillales* in different quantities (Supplementary Figure S2), the most highly expressed protein did not afford the highest reporter protein expression in the functional assay (Figure 2A). The observed differences in induction of the reporter are most likely due to a combination of different protein expression levels and different affinities of the AcoR variants for the DNA binding sites.

Next, we fused different transactivation domains, namely VPR, VP16 and VP16_f-type_ C- and N-terminally to AcoR to screen for the best fold induction (Figure 2B). We then placed VP16_f-type_-AcoR under the control of the different constitutive promoters mPGK, hSV40 and hCMV and evaluated SEAP expression (Figure 2C). To optimize the reporter plasmid, we constructed plasmids with different spacers between the minimal promoter and the AceA binding sequence (Figure 2D), or with one to four tandem repeats of the binding sequence (Figure 2E), or with different minimal promoters (Figure 2F). Increasing the distance from the binding sequence to the minimal promoter, as well as increasing the number of binding sequences increased the expression level, but led to lower fold changes due to higher basal expression of the reporter protein. Among the different minimal promoters tested, we selected hCMV_min_ for the follow-up experiments, as it showed the highest expression level of the reporter protein together with good fold induction.

Finally, we transfected eight different mammalian cell lines with the best-performing acetoin gene switch to evaluate its broader applicability. All the cell lines tested showed increased SEAP production upon exposure to acetoin (Figure 2G).

In order to examine the functionality of the acetoin-based gene switch in combination with another flavor-based gene switch, the vanillic acid VanR-VP16 switch OFF system, we co-expressed AceA, VanR-VP16 and two reporter proteins, SEAP under the control of AceA and nano luciferase (NLuc) under the control of VanR, in the same cells. With increasing concentrations of both flavor molecules (vanillic acid and acetoin) the expression of NLuc decreased while the expression of SEAP increased, demonstrating the compatibility of the two flavor-based transcriptional regulation systems (Figure 2H). Overall SEAP expression is reduced due to higher protein loads and potential squelching when both systems are used simultaneously, as both systems rely on the acidic viral transactivator VP16 (24,25).

### Generation of transgenic stable cell line for the acetoin gene switch

**Figure 3.**
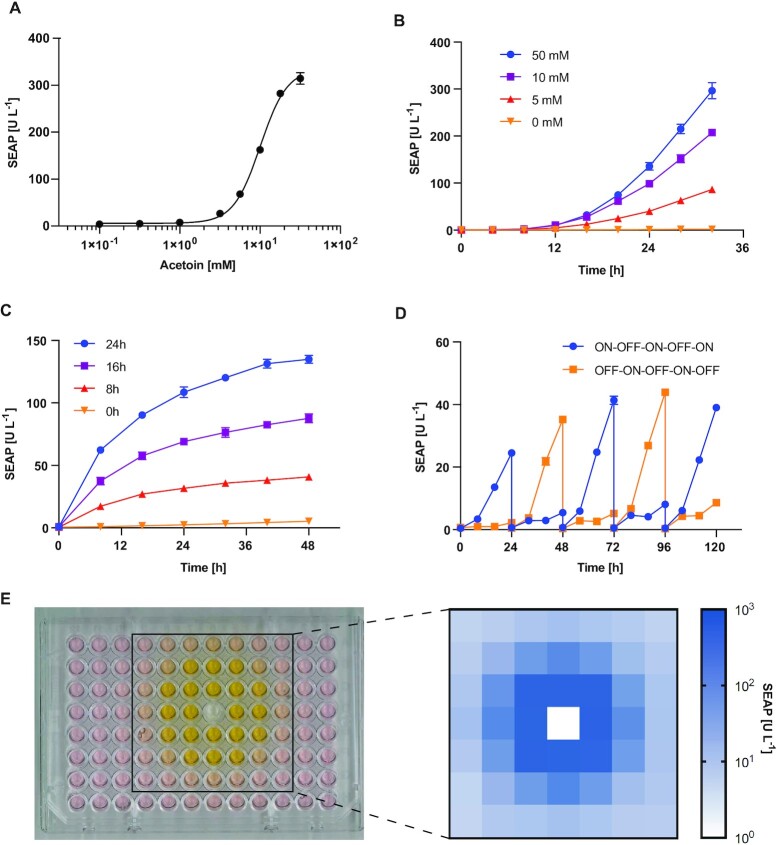
(**A**) Characterization of the SEAP levels produced by the stable monoclonal cell line HEK-AIGES when cultured in the presence of different acetoin concentrations. (**B**) Activation dynamics after exposure of HEK-AIGES cells to acetoin at different concentrations (0, 5, 10 and 50 mM). SEAP expression was monitored over a period of 32 h following the exposure to acetoin, with a sampling interval of 4 h. (**C**) HEK-AIGES cells were exposed to 10 mM acetoin for 0, 8, 16 and 24 h before the medium was exchanged to standard cell culture medium without acetoin, and SEAP expression was monitored over a period of 48 h at intervals of 8 h. (**D**) To test for reversibility, HEK-AIGES cells were exposed to acetoin (10 mM) or no acetoin for 8 h before the medium was exchanged. Cells were grown for an additional 16 h, then trypsinized and reseeded into fresh medium to maintain a stable cell number of 15 000 cells per well of a 96-well plate. Reseeding as well as media changes removed all SEAP in the medium, resetting the SEAP levels. The medium in which the cells were reseeded either contained acetoin (10 mM) or no acetoin. After 8 h the medium was exchanged again. This was repeated over a period of 120 h using two different protocols, starting either with acetoin (ON-OFF-ON-OFF-ON) or no acetoin (OFF-ON-OFF-ON-OFF). Samples were taken every 8 h. (**E**) Picture and heatmap of SEAP expression in a 96-well plate after volatile induction. Solid acetoin (10 mg) was placed in one dry well of a 96-well plate while other wells contained HEK-AIGES cells. SEAP level in each well was measured 24 h after acetoin addition. All experiments were performed in triplicate and repeated at least three times.

We established a HEK293T cell population with both components of the acetoin-inducible gene switch (P_mPGK_-AceA (pAB800) and O_AceA_P_hCMVmin_-SEAP (pAB801)) stably integrated in their genome using the Sleeping Beauty transposase system (20). Monoclonal cell lines were isolated and screened to select the one (HEK-AIGES) with the highest induced expression and lowest basal expression of the reporter protein SEAP (Supplementary Figure S3). We measured the dose-response curve of the HEK-AIGES cell line in response to acetoin (Figure 3A). The response was approximately linear between 3 mM and 33 mM with an EC50 of 10.22 mM. Cells cultured with different acetoin concentrations in this range showed no significant differences in cell viability, confirming that acetoin is not cytotoxic in this range (Supplementary Figure S4). To examine the time-dependence of reporter expression after exposure to acetoin, we measured SEAP expression over a period of 32 h after exposure to acetoin. SEAP production was observed after 8 h, and was significantly upregulated between 12 and 16 h post exposure (Figure 3B). Off-kinetics of the HEK-AIGES cell line was tested by exposing the cells to acetoin (10 mM) for 0, 8, 16 or 24 h. After the exposure, the medium was exchanged to acetoin-free medium and reporter gene production was measured over a period of 48 h (Figure 3C). Cells were co-transfected with a second reporter gene (NLuc) controlled by the constitutive promotor SV40 to ensure the off-kinetic observations were not affected by loss of cell viability over time due to overgrowth of the cells (Supplementary Figure S5). The overall induction level was positively correlated with the induction time.

To assess the reversibility of the gene switch, we repeatedly cultured the stable monoclonal cell line in the presence (ON) or absence (OFF) of acetoin for 8 h, then changed the medium to acetoin-free medium and followed SEAP expression for the next 16 h. After each 24-h cycle, we reseeded the cells to maintain a stable cell number before starting the next cycle. The results of five successive 24-h cycles using acetoin ON-OFF-ON-OFF-ON and OFF-ON-OFF-ON-OFF patterns are shown in Figure 3D. The results confirm that expression of SEAP can be reversibly controlled over multiple cycles.

Finally, we assessed whether AIGES cells would also be responsive to acetoin vapor generated by diffusion of solid acetoin. We placed acetoin powder in a middle well of 96-well plates. The acetoin spontaneously diffused and induced SEAP expression in cells seeded in the surrounding wells, in a distance-dependent manner (Figure 3E).

### *In vivo* performance of the AIGES to treat T1DM.

**Figure 4.**
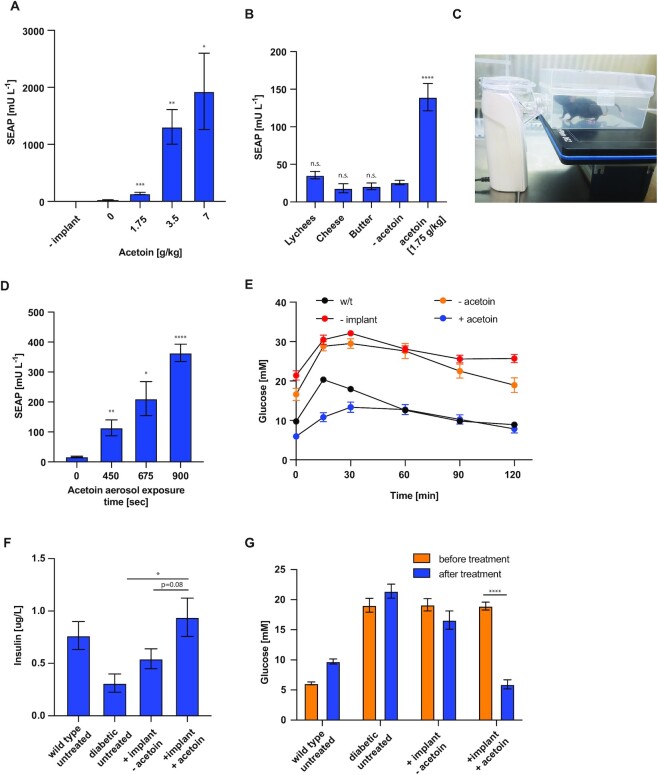
(**A**) SEAP levels in the bloodstream of wild-type mice with intraperitoneally implanted encapsulated HEK-AIGES cells following acetoin administration. Acetoin was delivered via oral gavage at the indicated doses. Mice without HEK-AIGES cell implants were used as negative controls. (**B**) SEAP production when mice were fed lychees, cheese or butter, which naturally contain acetoin. The lowest functional dosage of oral gavage-applied acetoin was used as a positive control. (**C**) Image showing the setup for inhaler-based induction. (**D**) Inhaler-based *in vivo* induction was conducted by vaporizing an acetoin containing solution (600 mg/ml) with repeated intervals of switching the inhaler on for 150 s and off for 150 s, for a total time of 900, 1350 and 1800 s, resulting in induction times of 450, 675 and 900 s. Waiting times were included to minimize oversaturation of the air with moisture. (**E**) Glucose tolerance test was performed by administering 1.5 g/kg glucose solution i.p. and the glycemia profile was generated by monitoring blood glucose levels over a period of 120 min. (**F**) Insulin levels were measured 24 h after acetoin treatment by assessing the serum insulin levels with an ELISA kit. (**G**) Blood glucose levels were measured before and 24 h after acetoin treatment. For panels (E), (F) and (G) acetoin administration was performed with the 900-s inhaler protocol. All experiments were performed twice with 8 mice per treatment group and 6 mice per control group. To test for significance, statistical analysis using one-way ANOVA was performed comparing all datasets of one representative biological replicate to the uninduced dataset (0 mM acetoin) in panels A–D.

To test the potential therapeutic applicability of the acetoin-inducible gene network, we injected alginate-encapsulated HEK-AIGES cells intraperitoneally (i.p.) in mice and measured the SEAP levels in the bloodstream in response to administration of acetoin by different means. Mice treated with an acetoin solution delivered via oral gavage showed increased blood SEAP levels in a dose-dependent manner (Figure 4A). To examine the kinetics of our gene switch, we examined acetoin depletion (Supplementary Figure S6), as well as SEAP and insulin kinetics after a single dose of acetoin [5 mg/kg] (Supplementary Figure S7). The intake of different acetoin-containing foods, namely lychees (26), cheddar cheese (27,28) and butter (29), did not activate SEAP expression (Figure 4B). Cell implants were also tested at three and five days after injection to check the longevity of the encapsulated cells (Supplementary Figure S8). Cells retrieved after being implanted were compared to cells cultured in vitro (Supplementary Figure S9) and inflammation markers were measured in mouse serum at five days after implantation (Supplementary Figure S10). No loss in functionality or signs of inflammation were observed. Mice exposed to acetoin aerosol generated by a portable inhaler containing an acetoin-rich solution (Figure 4C) for 450, 675 or 900 s showed an exposure time-dependent increase of blood SEAP levels (Figure 4D). In order to test the applicability of inhaler-based induction of the AIGES system in a disease model, we engineered a stable cell line with acetoin-responsive insulin production and chose the best monoclonal cell line (AIGES_Ins_) based on basal and total expression (Supplementary Figure S11) and tested for dose-dependent insulin production capacity (Supplementary Figure S12). We then injected type-I diabetic mice with encapsulated AIGES_Ins_ cells and exposed them to an acetoin-containing aerosol generated by an inhaler. Mice exposed to acetoin aerosol showed lower initial and peak glucose levels, as well as a faster decrease in blood glucose level after the spike in GTT assay, resembling the behavior seen in the non-diabetes control group (Figure 4E). To evaluate the full capacity of the AIGES_Ins_ cells we induced mice by exposure to acetoin aerosol for 900 s, which resulted in high insulin levels (Figure 4F) and corresponding low fasting-glucose levels (Figure 4G) at 24 h post treatment.

## DISCUSSION

There have been major advances in cell-based therapies in recent years, with several engineered T cell therapies approved to treat blood cancers, and many more in the pipeline (30–34). Some of these therapies need to be controlled by an external trigger to increase their safety. Ideally, a gene regulation system for cell-based therapies would be orthogonal and finely tunable by a non-toxic inducer that has a short half-life time *in vivo* and is physiologically inert and/or clinically licensed. While clinically licensed drugs are designed to have therapeutic effects (35–37), non-orthogonal inducers such as vitamins (38) and metabolites such as amino acids (39,40) have to be used at concentrations well above physiological levels to ensure control over gene expression. However, prolonged exposures to toxic or physiologically unnatural inducer levels pose the risk of severe side effects. To increase patient compliance and to avoid the need for medically trained persons to administer the inducer, a simple mode of administration of the inducer is essential for cell-based therapy.

Inhalation is an ancient route of drug administration. The first evidence of inhalation therapy was traced back 4000 years in India (41), but it was not until the middle of the 19th century when its significance was discovered for the treatment of lung diseases with the invention of the glass bulb nebulizer (41). Today, inhalers are integral to today's treatment options for asthma or chronic obstructive pulmonary disease (42). Indeed, many products are now commercially available to ease the administration of a drug or small molecule, including nicotine inhalers for nicotine reduction treatment (43).

The key to a successful implementation of gene- or cell-based therapies is to increase not only the patient's life expectancy, but also quality of life. As these therapies replace lost functionality, such as the expression of proteins or peptide hormones, a simple means of controlling their expression is critical, as it will be a regular part of the patient's life. In addition, it is of great importance that engineered cells are not triggered to produce the therapeutic protein unintentionally by any external stimulus. This is the main drawback of physically induced systems based on triggers such as heat, light or sound. Even though induction of these systems is very simple, avoiding unintended stimulation would have a drastic impact on the patient's life. Controlling gene expression via inhalation is therefore attractive. Previous inducible gene regulatory systems based on gaseous inducers are either too toxic for *in vivo* use (e.g. acetaldehyde (44)) or were designed to react to external stimuli present in cosmetic products or consumer goods used in everyday life (e.g. spearmint essential oil or chewing gum containing spearmint (2)), and therefore require constant attentiveness.

In contrast, our AIGES system is characterized by high inducibility and tight control without unspecific induction, combined with an easy route of trigger administration, and thus could be a very attractive option for future gene- and cell-therapies. The use of an inducer molecule not available from natural sources is advantageous, though relatively large amounts of the inducer are needed to activate gene transcription. One approach to enable good activation with a smaller quantity of inducer would be to select the location of the implant for optimal induction properties. Further studies are also needed to examine cell implant survival, in particular to avoid fibrotic overgrowth, a typical foreign-body response (45,46).

Given that the acetoin inducer is inexpensive and is already an FDA-approved food additive, AIGES could be a useful tool to regulate gene expression for therapeutic protein production in biphasic production processes where the cell expansion phase is decoupled from the protein production phase (47). The potential of AIGES for use in cell-based therapies was confirmed here in a proof-of-concept experiment using a type-1 diabetes mouse model, in which it effectively normalized blood glucose levels.

## DATA AVAILABILITY

All plasmids and data generated in this study are available on request. Requests for materials should be made to the corresponding author.

## Supplementary Material

gkac1256_Supplemental_File
