# Supplementary material for: Controlling therapeutic protein expression via inhalation of a butter flavor molecule
Source: Nucleic Acids Res. 2023 Jan 10;51(5):e28. doi: 10.1093/nar/gkac1256 (PMC10018347; doi:10.1093/nar/gkac1256)
Supplement: gkac1256_Supplemental_File [file gkac1256_supplemental_file.pdf]

Supplementary Material

Controlling therapeutic protein expression via inhalation of a butter flavor molecule

Adrian Bertschi[1], Bozhidar-Adrian Stefanov[1], Shuai Xue[1], Ghislaine Charpin-El Hamri[2], Ana Palma Teixeira[1], Martin Fussenegger*[1,3]

[1]Department of Biosystems Science and Engineering, ETH Zurich, Mattenstrasse 26, CH-4058 Basel, Switzerland.
[2]Département Génie Biologique, Institut Universitaire de Technologie, Université Claude Bernard, Lyon 1 Villeurbanne Cedex F-69622, France
[3]University of Basel, Faculty of Science, Mattenstrasse 26, CH-4058 Basel, Switzerland.
*Corresponding author. Email: martin.fussenegger@bsse.ethz.ch

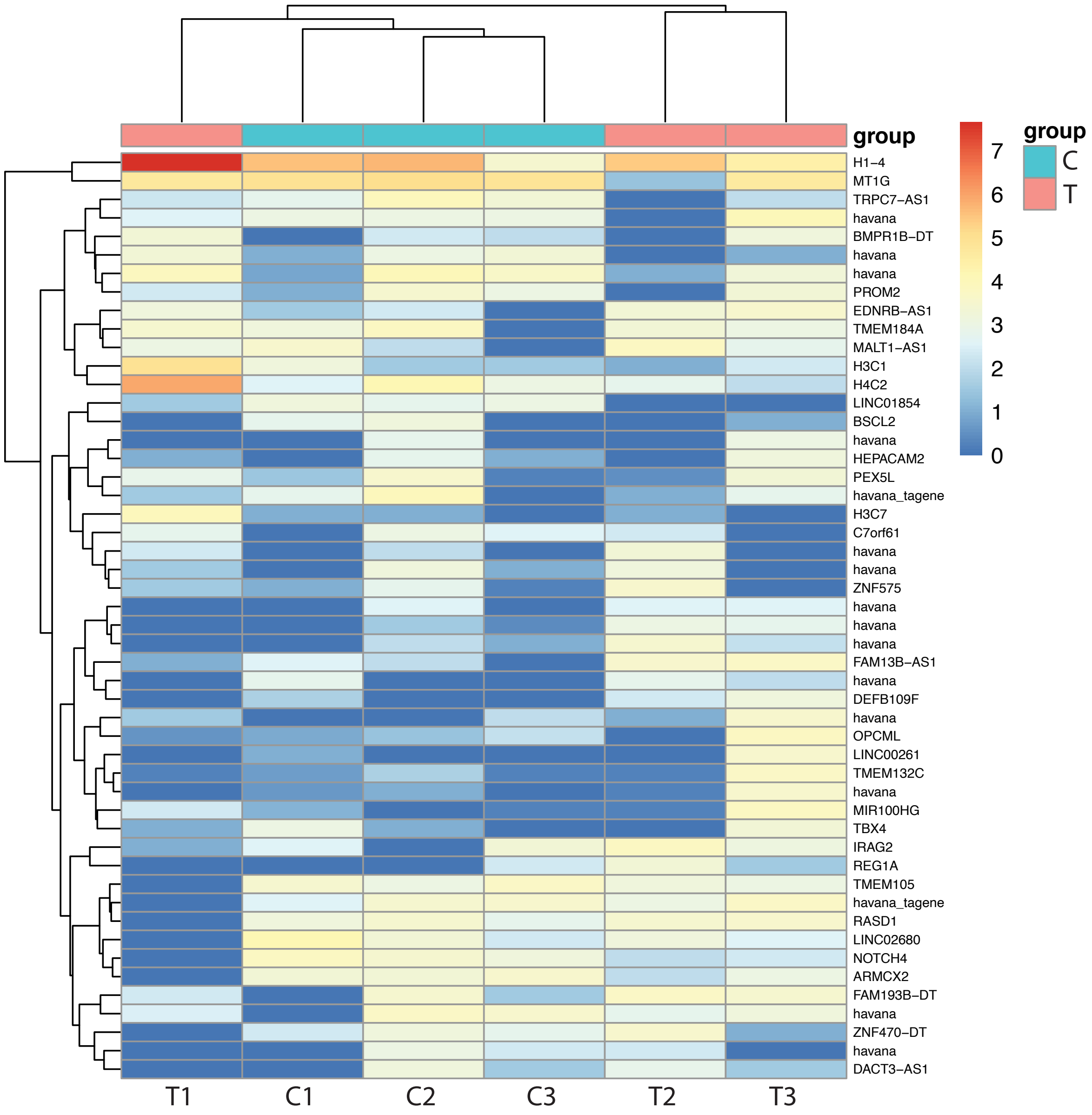

Figure S1: RNA-seq data of HEK293T cells untreated and treated with acetoin [10 mM]. For the 50 genes with top variance of expression, no clear clustering is seen on a heatmap of log2 of number of reads. Since overall fold changes and log2 of number of reads are low, there appears to be no marked difference between treated cells (T1-3) and untreated control (C1-3). The RNAseq data presented in this figure have been deposited in NCBI's Gene Expression Omnibus (Edgar et al., 2002) and are accessible via GEO Series accession number GSE217626 (https://www.ncbi.nlm.nih.gov/geo/query/acc.cgi?acc=GSE217626).

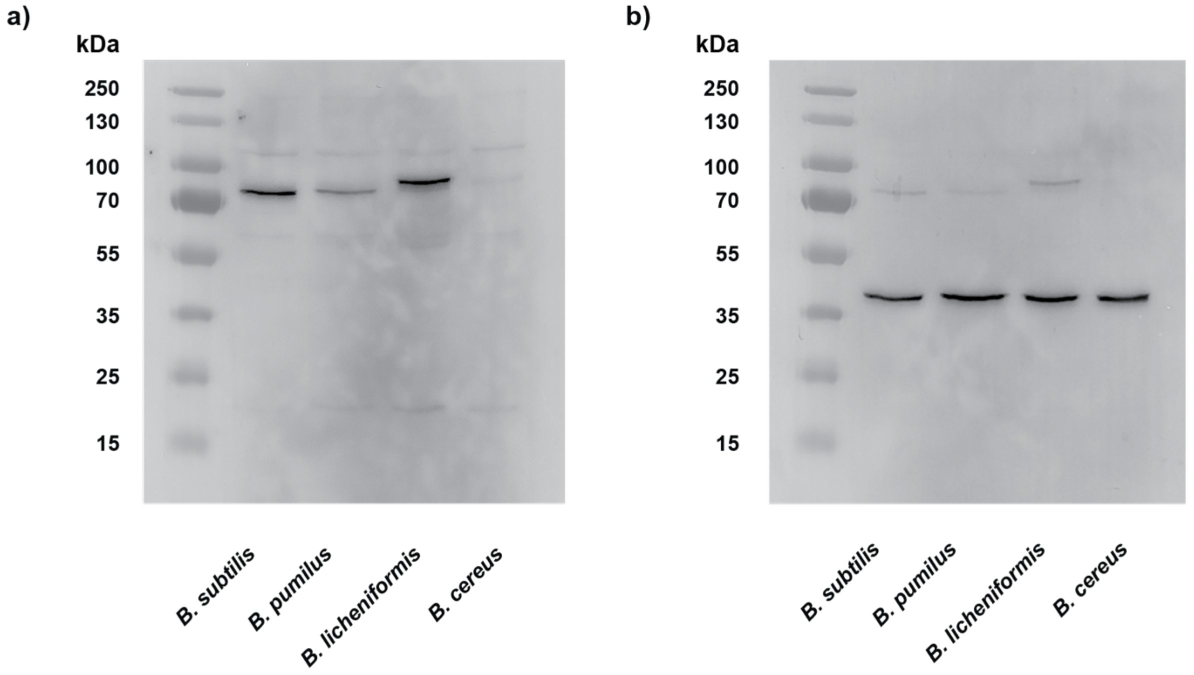

Figure S2: Western-blot analysis of AcoR proteins from different *Bacillales* in HEK293T cells. a) Different expression levels of FLAG tagged AcoR-VP16$_{\text{f-type}}$ with AcoR derived from *B. subtilis*, *B. pumilus, B. licheniformis* or *B. cereus* (~77 kDa) and b) actin (45 kDa) loading control. AcoR protein sequences from the different *Bacillales* show different AcoR production quantities in HEK cells with AcoR$_{licheniformis}$ being the most highly expressed protein and AcoR$_{cereus}$ the least highly expressed variant among the four tested.

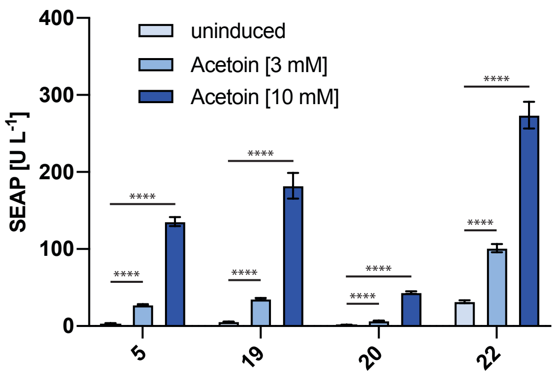

Figure S3: SEAP expression by the four best-performing monoclonal cell lines derived from the polyclonal cell line sAB05. The four monoclonal cell lines were selected out of 23 colonies expanded from wells of one 96-well plate, each seeded with a single cell. Cell line 19, which showed high SEAP expression and the largest fold-change in the presence of acetoin, was selected for all further experiments and is referred to as HEK-AIGES.

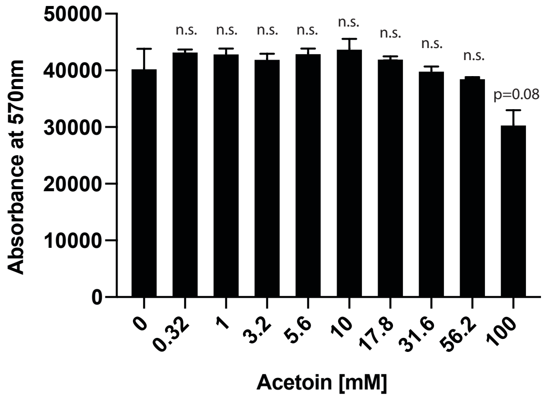

Figure S4: Cell viability assay. HEK-293T cells were cultured for 24 hours in standard cell culture medium containing different concentrations of acetoin ranging from 0 to 100 mM. After 24 hours, cell viability was evaluated by resazurin assay for 2 hours. The absorbance at 570 nm was measured. One-way ANOVA was performed comparing each bar to the negative control without acetoin.

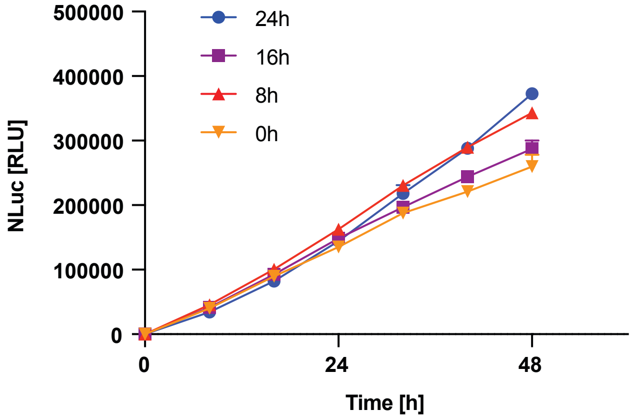

Figure S5: Constitutive NLuc production of HEK-AIGES cells co-transfected with SV40 NLuc over a period of 48 hours to monitor protein production capability over time as a control for the AIGES off-switching characteristics in Figure 3c. HEK-AIGES cells were seeded in a 6-well plate and induced for either 0, 8 16 or 24 hours with acetoin (10 mM). The medium was exchanged at timepoint 0 to fresh medium without acetoin, and reporter protein production was measured over 48 hours.

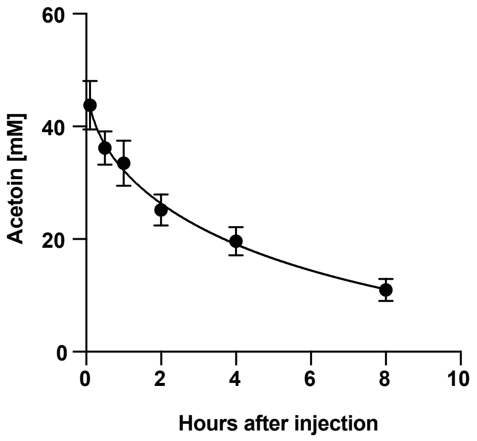

Figure S6: Pharmacokinetics of acetoin in mice. Mice were injected with 5 g/kg acetoin i.p. and blood serum samples were collected at the indicated time points after injection. Acetoin levels in the blood serum samples were determined using the HEK-AIGES cell line by adding 20% serum to the cells and calculating the acetoin concentration in the serum based on a calibration curve.

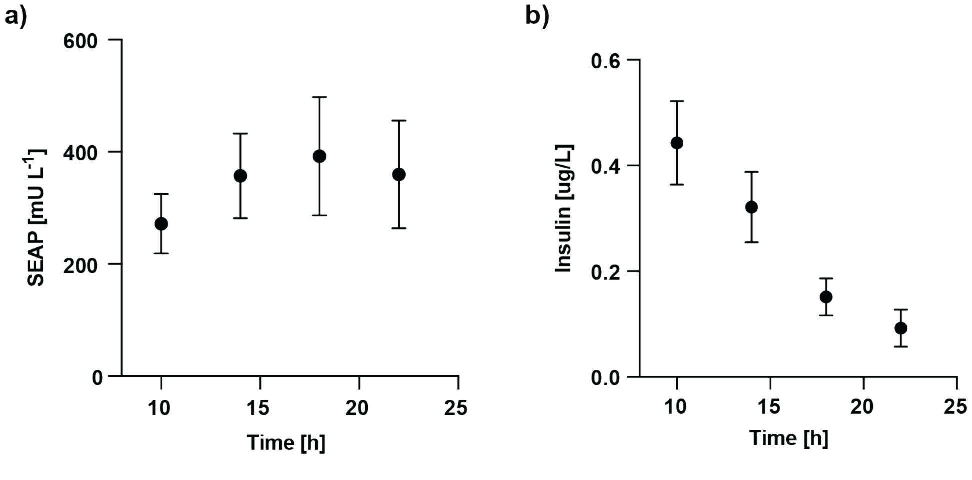

Figure S7: Off kinetics of HEK-AIGES$_{ins}$ cell implants after induction by acetoin. SEAP a) and insulin b) levels were measured in the blood of type 1 diabetic mice every 4 hours starting at 10 hours after acetoin administration. SEAP, which is more stable, increases until 18 hours

after induction, while the insulin levels decrease continuously during this period, returning to a normal fasting level 18 hours after induction.

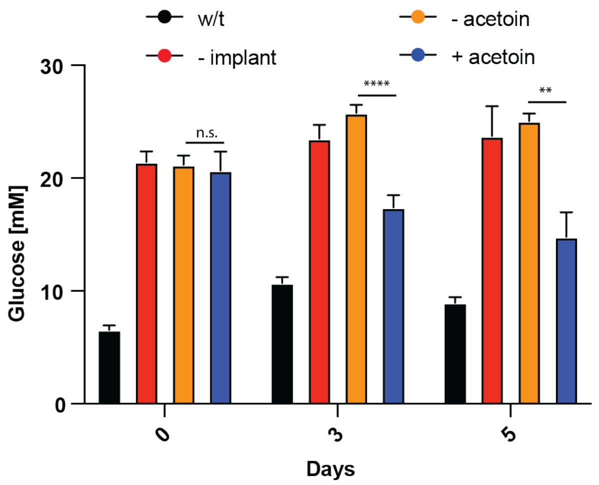

Figure S8: HEK-AIGES$_{ins}$ cells were encapsulated and injected i.p. into diabetic mice. To assess long-term functionality, blood glucose levels were measured at 3 and 5 days after injection of the capsules. While blood glucose levels of the negative control and the uninduced group remained high, the induced group showed significantly reduced fasting glucose levels at 3 and 5 days after injection of the capsules. Mice were administered acetoin o.g. 16 hours prior to blood glucose measurements and were fasted overnight. Each group consists of 8 mice.

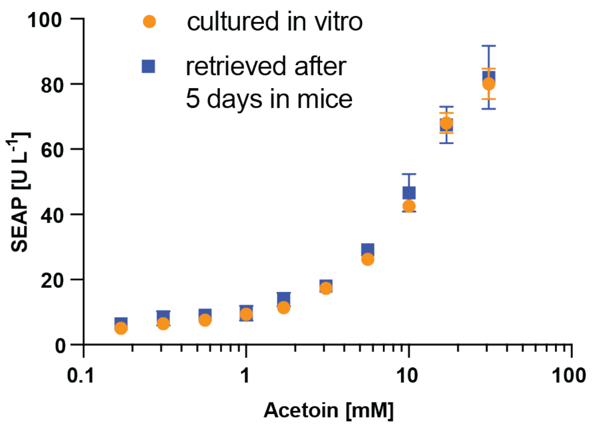

Figure S9: Comparison of acetoin-inducible SEAP production by HEK-AIGES$_{ins}$ cells before implantation and at 5 days after implantation in mice. The dose-dependent response of HEK-AIGES$_{ins}$ cells retrieved after being encapsulated and transplanted into mice for 5 days is not significantly different from that of HEK-AIGES$_{ins}$ cells kept in *in vitro* culture.

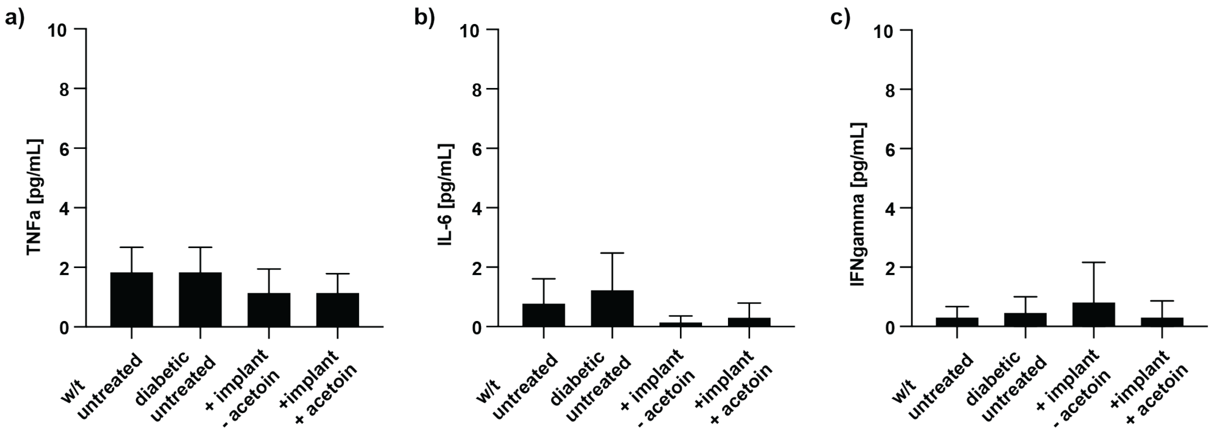

Figure S10: Inflammation markers in the blood of mice. To examine whether there is an inflammatory response towards the encapsulated cells, we analyzed the concentrations of a) TNFα, b) IL-6 and c) IFNγ in blood samples of mice at 5 days after implantation, using commercial ELISA kits.

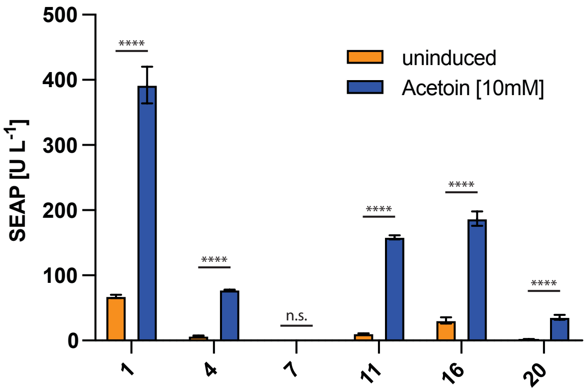

Figure S11: Stable monoclonal cell lines producing SEAP-p2a-insulin were tested for basal and induced production of the reporter gene. We selected six monoclonal cell lines for further

analysis from a total of 28 colonies, expanded in 96-well plates previously seeded with an average of one cell per well. The stable monoclonal cell line 1 showed the highest total expression with a similar fold switching to other monoclonal cell lines, and was selected for all further experiments. Cell line 1 is referred to as HEK-AIGES$_{ins}$.

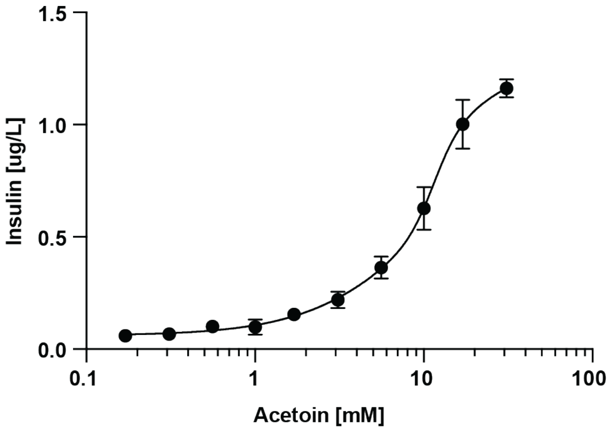

Figure S12: Dose-dependent insulin production by the stable monoclonal HEK-AIGES$_{ins}$ cell line. Insulin production increases 20-fold from the lowest to the highest acetoin concentration, while the EC50 lies at 9.8 mM.

Table S1: Plasmids used and constructed in this work

Plasmid Name	Description	Reference / Accession number
pDF101	Inert filler plasmid bearing a bacterial T7 promoter driving an inactive ribozyme. <br>(P_{T7}-SpAL-sTRSVac)	(Ausländer et al., 2016)
SEAP2-Control	SV40-driven SEAP expression plasmid <br>(P_{SV40}-SEAP-pA).	Clonetech
pSP16	CRE-driven SEAP reporter protein expression vector. <br>(P_{CREm}-$P_{gl4.23}$-SEAP-pA_{SV40}).	(Saxena et al., 2016)
pFOX8	CMV-driven YPet expression plasmid <br>(P_{hCMV}-Ypet-pA).	(Haellman, Strittmatter, et al., 2021)
pMM328	PGK-driven SEAP expression plasmid <br>(P_{mPGK}-SEAP-pA).	(Chassin et al., 2019)
$mCherry_{mFT}$	$mCherry_{mFT}$ expression plasmid <br>(P_{hCMV}-$mCherry_{mFT}$-pA_{bGH}).	(Subach et al., 2009)
pANA268	Mammalian SEAP reporter protein expression vector from the $P_{tata\text{-}T7}$ minimal promoter. <br>($P_{tata\text{-}T7}$-SEAP-pA_{bGH})	Teixeira et al. unpublished

pBS237	Mammalian SEAP reporter protein expression vector from the $P_{YB\text{-}tata}$ minimal promoter.<br><br>($P_{YB\text{-}tata}$-SEAP-pA_{bGH})	Stefanov et al. unpublished
pBS828	Sleeping Beauty-specific transposon encoding $P_{hCMV*\text{-}1}$-driven SEAP and mINS expression as well as constitutive P_{RPBSA}- driven YPet and PuroR expression.<br><br>(ITR-$_{PhCMV*\text{-}1}$-SEAP-P2A-mINS-pA_{bGH}-P_{PRPBSA}-EGFP-P2A-ZeoR-pA_{bGH}-ITR)	(Stefanov et al., 2021)
pTS395	P_{hCMV}-driven Sleeping Beauty transposase mammalian expression vector.<br><br>(P_{hCMV}-SB100- pA_{bGH}).	(Haellman, Strittmatter, et al., 2021)
pTS1015	SEAP expression vector with a minimal version of the CMV promoter.<br><br>($P_{hCMVmin}$-SEAP-pA_{bGH})	Strittmatter et al. unpublished
pTS1214	p2a expression plasmid.<br><br>(P_{hCMV}-p2a-pA_{bGH}).	Strittmatter et al. unpublished
pTS2339	Stable integration vector via Sleeping Beauty transposase including a mRuby2 and PuroR resistance gene<br><br>(ITR-P_{RPBSA}-mRuby2-p2a-PuroR-pA_{p9}-ITR)	Strittmatter et al. unpublished
pTS2341	Stable integration vector via Sleeping Beauty transposase including a PuroR resistance gene<br><br>(ITR-P_{RPBSA}-PuroR-pA_{p9}-ITR)	Strittmatter et al. unpublished
pTS2346	Stable integration vector via Sleeping Beauty transposase including a BlastR resistance gene<br><br>(ITR-P_{RPBSA}-BlastR-pA_{p9}-ITR)	Strittmatter et al. unpublished
pTS2366	$VP16_{f\text{-}type}$ transactivation domain expression plasmid.	Strittmatter et al.

	(P_{hCMV}-VP16$_{f\text{-}type}$-pA$_{bGH}$).	unpublished
pTS2367	VP16 transactivation domain expression plasmid.<br><br>(P_{hCMV}-VP16-pA$_{bGH}$).	Strittmatter et al. unpublished
pVH15	VPR transactivation domain expression plasmid.<br><br>(P_{hCMV}-VPR-pA$_{bGH}$).	Haellman et al. unpublished
pVH21	$P_{hEF1\alpha}$-driven expression vector<br><br>($P_{hEF1\alpha}$-MCS-pA).	(Haellman, Strittmatter, et al., 2021)
pVH254	Doxycycline-inducible SEAP reporter protein expression vector.<br><br>(O_{TetO7}-P_{min}-SEAP-pA).	(Haellman, Saxena, et al., 2021)
pVH261	Constitutive expression vector VanR-FL$_1$-VP16 containing a flexible GGGS-linker.<br><br>(P_{mPGK1}-VanR-FL$_1$-VP16- pA$_{bGH}$)	(Haellman, Saxena, et al., 2021)
pVH294	Vanillic acid-inducible Igk-nLuc reporter protein expression vector.<br><br>(O_{VanO2}-$P_{CMVmin\text{-}1}$-SP-Nluc-pA).	(Haellman, Saxena, et al., 2021)
pAB001	AcoR from *Bacillus subtilis* expression plasmid<br><br>AcoR was restricted from ACOR_BACSU (O31551, uniprot), human optimized and synthesized via TWIST with the flanking regions of (5' CATGACTAGTGGTGGTTCTGGT 3') N-terminal and (5' GCTAGCGGATCCTCAG 3') C-terminal, using *SpeI* and *BamHI* and inserted into the corresponding sites of pFOX8.	This work

	(P_{hCMV}-AcoR-pA_{bGH}).	
pAB002	AcoR from *Bacillus subtilis* expression plasmid<br><br>VP16$_{f\text{-}type}$ was restricted using *EcoRI* and *NheI* from pTS2366 and inserted into *EcoRI SpeI* restricted pAB001.<br><br>(P_{hCMV}-VP16$_{f\text{-}type}$-AcoR-pA_{bGH}).	This work
pAB003	AcoR from *Bacillus subtilis* expression plasmid<br><br>P_{mPGK} was restricted using *MluI* and *EcoRI* from pMM328 and inserted into the corresponding sites of pAB002<br><br>(P_{mPGK}-VP16$_{f\text{-}type}$-AcoR-pA_{bGH}).	This work
pAB004	AcoR from *Bacillus subtilis* expression plasmid<br><br>P_{SV40} was restricted using *MluI* and *EcoRI* from SEAP2-Control and inserted into the corresponding sites of pAB002<br><br>(P_{SV40}-VP16$_{f\text{-}type}$-AcoR-pA_{bGH}).	This work
pAB005	AcoR-inducible mammalian SEAP reporter protein expression vector with operator sequence from *Bacillus Licheniformis* expression plasmid<br><br>AcoR was restricted from Q65MC7_BACLD (Q65MC7, uniprot), human optimized and synthesized via TWIST with the flanking regions of (5' CATGGAATTCACCATGACTAGTGGTGGTTCTGGT 3') N-terminal and (5' GCTAGCGGATCCTCAG 3') C-terminal, using *EcoRI* and *NheI* and inserted into pTS2366 which was restricted using *EcoRI* and *SpeI.*<br><br>(P_{mPGK}-AcoR-VP16$_{f\text{-}type}$-pA_{bGH}).	This work

pAB006	AcoR-inducible mammalian SEAP reporter protein expression vector with operator sequence from *Bacillus Pumilus* expression plasmid<br><br>AcoR was restricted from A0REX6_BACAH (A0REX6, uniprot), human optimized and synthesized via TWIST with the flanking regions of (5' CATGGAATTCACCATGACTAGTGGTGGTTCTGGT 3') N-terminal and (5' GCTAGCGGATCCTCAG 3') C-terminal, using *EcoRI* and *NheI* and inserted into pTS2366 which was restricted using *EcoRI* and *SpeI.*<br><br>(P_{mPGK}-AcoR-VP16$_{f\text{-}type}$-pA$_{bGH}$).	This work
pAB007	AcoR-inducible mammalian SEAP reporter protein expression vector with operator sequence from *Bacillus cereus* expression plasmid<br><br>AcoR was restricted from Q63AI3_BACCZ (Q63AI3, uniprot), human optimized and synthesized via TWIST with the flanking regions of (5' CATGGAATTCACCATGACTAGTGGTGGTTCTGGT 3') N-terminal and (5' GCTAGCGGATCCTCAG 3') C-terminal, using *EcoRI* and *NheI* and inserted into pTS2366 which was restricted using *EcoRI* and *SpeI.*<br><br>(P_{mPGK}-AcoR-VP16$_{f\text{-}type}$-pA$_{bGH}$).	This work
pAB008	AcoR-inducible mammalian SEAP reporter protein expression vector with operator sequence from *Bacillus subtilis* AcoO P_{CMVmin} SEAP reporter expression vector<br><br>O_{AcoR} was obtained through annealing and phosphorylation of oAB001 (CGCGTCTCGAGCGAGACAAATGAATCAGTTTGAGACAAAACGAGACACACGTCTCAAACTGa) and oAB002 (tcgatCAGTTTGAGACGTGTGTCTCGTTTTGTCTCAAACTGATTCATTTGTCTCGCTCGAGA) and ligation into *MluI XhoI* restricted pTS1015.<br><br>(P_{OAcoR}-$P_{hCMVmin}$-SEAP- pA$_{bGH}$).	This work
pAB009	AcoR from *Bacillus subtilis* expression plasmid	This work

	$VP16_{f\text{-}type}$ was restricted using *SpeI* and *HindIII* from pTS2366 and inserted into *NheI HindIII* restricted pAB001.<br><br>(P_{hCMV}-AcoR-$VP16_{f\text{-}type}$-pA_{bGH}).	
pAB010	AcoR from *Bacillus subtilis* expression plasmid<br><br>VP16 was restricted using *EcoRI* and *NheI* from pTS2367 and inserted into *EcoRI SpeI* restricted pAB001.<br><br>(P_{hCMV}-VP16-AcoR-pA_{bGH}).	This work
pAB011	AcoR from *Bacillus subtilis* expression plasmid<br><br>VP16 was restricted using *SpeI* and *HindIII* from pTS2367 and inserted into *NheI HindIII* restricted pAB001.<br><br>(P_{hCMV}-AcoR-VP16-pA_{bGH}).	This work
pAB012	AcoR from *Bacillus subtilis* expression plasmid<br><br>VPR was restricted using *EcoRI* and *NheI* from pVH15 and inserted into *EcoRI SpeI* restricted pAB001.<br><br>(P_{hCMV}-VPR-AcoR-pA_{bGH}).	This work
pAB013	AcoR from *Bacillus subtilis* expression plasmid<br><br>VPR was restricted using *SpeI* and *HindIII* from pVH15 and inserted into *NheI HindIII* restricted pAB001.<br><br>(P_{hCMV}-AcoR-VPR-pA_{bGH}).	This work

pAB014	AcoR from *Bacillus subtilis* expression plasmid<br><br>$P_{hEF1\alpha}$ was restricted using *MluI* and *EcoRI* from pVH21 and inserted into the corresponding sites of pAB002<br><br>($P_{hEF1\alpha}$-VP16$_{f\text{-}type}$-AcoR-pA$_{bGH}$).	This work
pAB015	AcoR from *Bacillus subtilis* expression plasmid<br><br>P_{mPGK} was restricted using *MluI* and *EcoRI* from pMM328 and inserted into the corresponding sites of pAB009.<br><br>(P_{mPGK}-AcoR-VP16$_{f\text{-}type}$-pA$_{bGH}$).	This work
pAB016	AcoR from *Bacillus subtilis* containing FLAG sequence expression plasmid<br><br>FLAG was obtained through annealing and phosphorylation of oAB121 (ctagtGGTGGTTCTGGTGACTACAAGGATGACGATGACAAGGCTAGCg) and oAB122 (gatccGCTAGCCTTGTCATCGTCATCCTTGTAGTCACCAGAACCACCa) and ligation into *NheI BamHI* restricted pAB015.<br><br>(P_{mPGK}-AcoR-VP16$_{f\text{-}type}$-FLAG-pA$_{bGH}$).	This work
pAB017	AcoR from *Bacillus Licheniformis* containing FLAG sequence expression plasmid<br><br>FLAG was obtained through annealing and phosphorylation of oAB121 (ctagtGGTGGTTCTGGTGACTACAAGGATGACGATGACAAGGCTAGCg) and oAB122 (gatccGCTAGCCTTGTCATCGTCATCCTTGTAGTCACCAGAACCACCa) and ligation into *NheI BamHI* restricted pAB005.<br><br>(P_{mPGK}-AcoR-VP16$_{f\text{-}type}$-FLAG-pA$_{bGH}$).	This work
pAB018	AcoR from *Bacillus Pumilus* containing FLAG sequence expression plasmid	This work

	FLAG was obtained through annealing and phosphorylation of oAB121 (ctagtGGTGGTTCTGGTGACTACAAGGATGACGATGACAAGGCTAGCg) and oAB122 (gatccGCTAGCCTTGTCATCGTCATCCTTGTAGTCACCAGAACCACCa) and ligation into *NheI BamHI* restricted pAB006.<br><br>(P_{mPGK}-AcoR-VP16$_{f\text{-}type}$-FLAG-pA$_{bGH}$).	
pAB019	AcoR from *Bacillus cereus* containing FLAG sequence expression plasmid<br><br>FLAG was obtained through annealing and phosphorylation of oAB121 (ctagtGGTGGTTCTGGTGACTACAAGGATGACGATGACAAGGCTAGCg) and oAB122 (gatccGCTAGCCTTGTCATCGTCATCCTTGTAGTCACCAGAACCACCa) and ligation into *NheI BamHI* restricted pAB007.<br><br>(P_{mPGK}-AcoR-VP16$_{f\text{-}type}$-FLAG-pA$_{bGH}$).	This work
pAB101	AcoR-inducible mammalian SEAP reporter protein expression vector with consensus sequence 1,2,1',2' $P_{hCMVmin}$ SEAP reporter expression vector<br><br>O_{AcoR} was obtained through annealing and phosphorylation of oAB003 (cGAGACAAAATGAGACATATGTCTCATTTTGTCTCa) and oAB004 (tcgatGAGACAAAATGAGACATATGTCTCATTTTGTCTCggtac) and ligation into *KpnI XhoI* restricted pTS1015.<br><br>(P_{OAcoR}-$P_{hCMVmin}$-SEAP- pA$_{bGH}$).	This work
pAB102	AcoR-inducible mammalian SEAP reporter protein expression vector with consensus sequence 1,2,1' $P_{hCMVmin}$ SEAP reporter expression vector<br><br>O_{AcoR} was obtained through annealing and phosphorylation of oAB005 (cGAGACAAAATGAGACATATGTCTCATTTgcatgaa) and oAB006	This work

	(tcgattcatgcAAATGAGACATATGTCTCATTTTGTCTCggtac) and ligation into *KpnI XhoI* restricted pTS1015.<br><br>($P_{OAcoR(1,2,1')}$-$P_{hCMVmin}$-SEAP- pA_{bGH}).	
pAB103	AcoR-inducible mammalian SEAP reporter protein expression vector with consensus sequence 1,2,2' $P_{hCMVmin}$ SEAP reporter expression vector<br><br>O_{AcoR} was obtained through annealing and phosphorylation of oAB007 (cGAGACAAAATGAGACATAgcatgaATTTTGTCTCa) and oAB008 (tcgatGAGACAAAATtcatgcTATGTCTCATTTTGTCTCggtac) and ligation into *KpnI XhoI* restricted pTS1015.<br><br>($P_{OAcoR(1,2,2')}$-$P_{hCMVmin}$-SEAP- pA_{bGH}).	This work
pAB104	AcoR-inducible mammalian SEAP reporter protein expression vector with consensus sequence 1,1',2' $P_{hCMVmin}$ SEAP reporter expression vector<br><br>O_{AcoR} was obtained through annealing and phosphorylation of oAB009 (cGAGACAAAATgcatgaTATGTCTCATTTTGTCTCa) and oAB010 (tcgatGAGACAAAATGAGACATAtcatgcATTTTGTCTCggtac) and ligation into *KpnI XhoI* restricted pTS1015.<br><br>($P_{OAcoR(1,1',2')}$-$P_{hCMVmin}$-SEAP- pA_{bGH}).	This work
pAB105	AcoR-inducible mammalian SEAP reporter protein expression vector with consensus sequence 2,1',2' $P_{hCMVmin}$ SEAP reporter expression vector<br><br>O_{AcoR} was obtained through annealing and phosphorylation of oAB011 (cgcatgaAAATGAGACATATGTCTCATTTTGTCTCa) and oAB012	This work

	(tcgatGAGACAAAATGAGACATATGTCTCATTTtcatgcggtac) and ligation into *KpnI XhoI* restricted pTS1015.<br><br>($P_{OAcoR(2,1',2')}$-$P_{hCMVmin}$-SEAP- pA_{bGH}).	
pAB106	AcoR-inducible mammalian SEAP reporter protein expression vector with consensus sequence 1,2 $P_{hCMVmin}$ SEAP reporter expression vector<br><br>O_{AcoR} was obtained through annealing and phosphorylation of oAB013 (cGAGACAAAATGAGACATAgcatgaATTTgcatgaa) and oAB014 (tcgattcatgcAAATtcatgcTATGTCTCATTTTGTCTCggtac) and ligation into *KpnI XhoI* restricted pTS1015.<br><br>($P_{OAcoR(1,2)}$-$P_{hCMVmin}$-SEAP- pA_{bGH}).	This work
pAB107	AcoR-inducible mammalian SEAP reporter protein expression vector with consensus sequence 1',2' $P_{hCMVmin}$ SEAP reporter expression vector<br><br>O_{AcoR} was obtained through annealing and phosphorylation of oAB015 (cgcatgaAAATgcatgaTATGTCTCATTTTGTCTCa) and oAB016 (tcgatGAGACAAAATGAGACATAtcatgcATTTtcatgcggtac) and ligation into *KpnI XhoI* restricted pTS1015.<br><br>($P_{OAcoR(1',2')}$-$P_{hCMVmin}$-SEAP- pA_{bGH}).	This work
pAB108	AcoR-inducible mammalian SEAP reporter protein expression vector with consensus sequence 1,2' $P_{hCMVmin}$ SEAP reporter expression vector<br><br>O_{AcoR} was obtained through annealing and phosphorylation of oAB017 (cGAGACAAAATgcatgaTAgcatgaATTTTGTCTCa) and oAB018	This work

	(tcgatGAGACAAAATtcatgcTAtcatgcATTTTGTCTCggtac) and ligation into *KpnI XhoI* restricted pTS1015.<br><br>($P_{OAcoR(1,2')}$-$P_{hCMVmin}$-SEAP- pA_{bGH}).	
pAB109	AcoR-inducible mammalian SEAP reporter protein expression vector with consensus sequence 2,1' $P_{hCMVmin}$ SEAP reporter expression vector<br><br>O_{AcoR} was obtained through annealing and phosphorylation of oAB019 (cgcatgaAAATGAGACATATGTCTCATTTgcatgaa) and oAB020 (tcgattcatgcAAATGAGACATATGTCTCATTTtcatgcggtac) and ligation into *KpnI XhoI* restricted pTS1015.<br><br>($P_{OAcoR(2,1')}$-$P_{hCMVmin}$-SEAP- pA_{bGH}).	This work
pAB110	AcoR-inducible mammalian SEAP reporter protein expression vector with consensus sequence 1,2' $P_{hCMVmin}$ SEAP reporter expression vector<br><br>O_{AcoR} was obtained through annealing and phosphorylation of oAB021 (cGAGACAAAATgcatgaTATGTCTCATTTgcatgaa) and oAB022 (tcgattcatgcAAATGAGACATAtcatgcATTTTGTCTCggtac) and ligation into *KpnI XhoI* restricted pTS1015.<br><br>($P_{OAcoR(1,1')}$-$P_{hCMVmin}$-SEAP- pA_{bGH}).	This work
pAB111	AcoR-inducible mammalian SEAP reporter protein expression vector with consensus sequence 1,2' $P_{hCMVmin}$ SEAP reporter expression vector<br><br>O_{AcoR} was obtained through annealing and phosphorylation of oAB023 (cgcatgaAAATGAGACATAgcatgaATTTTGTCTCa) and oAB024	This work

	(tcgatGAGACAAAATtcatgcTATGTCTCATTTtcatgcggtac) and ligation into *KpnI Xho*I restricted pTS1015.<br><br>($P_{OAcoR(2,2')}$-$P_{hCMVmin}$-SEAP- pA_{bGH}).	
pAB112	AcoR-inducible mammalian SEAP reporter protein expression vector with 1 x consensus sequence 1,2,1',2' $P_{hCMVmin}$ SEAP reporter expression vector.<br><br>O_{AcoR} was obtained through annealing and phosphorylation of oAB025 (cgcgtggtaccctcgagactcttcatacgttgGAGACAAAATGAGACATATGTCTCATTTTGTCTCa) and oAB026 (tcgatGAGACAAAATGAGACATATGTCTCATTTTGTCTCcaacgtatgaagagtctcgagggtacca) and ligation into *MluI XhoI* restricted pTS1015.<br><br>(P_{O1AcoR}-$P_{hCMVmin}$-SEAP- pA_{bGH}).	This work
pAB113	AcoR-inducible mammalian SEAP reporter protein expression vector with 2 x consensus sequence 1,2,1',2' $P_{hCMVmin}$ SEAP reporter expression vector.<br><br>O_{AcoR} was obtained through annealing and phosphorylation of oAB025 (c gcgtggtaccctcgagactcttcatacgttgGAGACAAAATGAGACATATGTCTCATTTTGTCTCa) and oAB026 (tcgatGAGACAAAATGAGACATATGTCTCATTTTGTCTCcaacgtatgaagagtctcgagggtacca) and ligation into *MluI XhoI* restricted pAB110.<br><br>(P_{O2AcoR}-$P_{hCMVmin}$-SEAP- pA_{bGH}).	This work
pAB114	AcoR-inducible mammalian SEAP reporter protein expression vector with 3 x consensus sequence 1,2,1',2' P $P_{hCMVmin}$ SEAP reporter expression vector.<br><br>O_{AcoR} was obtained through annealing and phosphorylation of oAB025 (c gcgtggtaccctcgagactcttcatacgttgGAGACAAAATGAGACATATGTCTCATTTTGTCTCa) and	This work

	oAB026 (tcgatGAGACAAAATGAGACATATGTCTCATTTTGTCTCcaacgtatgaagagtctcgagggtacca) and ligation into *MluI XhoI* restricted pAB111.<br><br>(P_{O3AcoR}-$P_{hCMVmin}$-SEAP- pA_{bGH}).	
pAB115	AcoR-inducible mammalian SEAP reporter protein expression vector with 4 x consensus sequence 1,2,1',2' $P_{hCMVmin}$ SEAP reporter expression vector.<br><br>O_{AcoR} was obtained through annealing and phosphorylation of oAB025 (c gcgtggtaccctcgagactcttcatacgttgGAGACAAAATGAGACATATGTCTCATTTTGTCTCa) and oAB026 (tcgatGAGACAAAATGAGACATATGTCTCATTTTGTCTCcaacgtatgaagagtctcgagggtacca) and ligation into *MluI XhoI* restricted pAB112.<br><br>(P_{O4AcoR}-$P_{hCMVmin}$-SEAP- pA_{bGH}).	This work
pAB800	Stable Sleeping Beauty integration plasmid bearing AcoR and a blasticidin resistance gene.<br><br>P_{hEF1a}-VP16$_{f\text{-}type}$-AcoR was restricted using *MluI* and *SpeI* form from pAB014 and AcoR was restricted using *SpeI* and *HindIII* from pAB001. Both were sequentially inserted into pTS1214 restricted with *MluI SpeI* and *NheI HindIII* to build the intermediate product pAB050 (P_{hEF1a}-VP16$_{f\text{-}type}$-AcoR-p2a-AcoR-pA_{bGH}). pAB050 was then restricted using *MluI* and *HindIII* and inserted in the corresponding site of pTS2346.<br><br>(ITR-P_{hEF1a}-VP16$_{f\text{-}type}$-AcoR-p2a-AcoR-pA_{bGH}-P_{RPBSA}-BlastR-pA_{p9}-ITR)	This work
pAB801	Stable Sleeping Beauty integration plasmid bearing the reporter vector driving SEAP expression, coupled to a fluorescent timer protein mCherry$_{mFT}$ via a p2a and a puromycin resistance gene.<br><br>P_{AcoO}-P_{CMVmin}-SEAP was restricted using *MluI* and *NheI* form pAB101 and mCherry$_{mFT}$ was restricted using *SpeI* and *BamHI* from mCherry$_{mFT}$. Both were sequentially inserted into pTS1214 restricted with *MluI SpeI* and *NheI BamHI* to build the intermediate product pAB051 (P_{AcoO}-P_{CMVmin}-SEAP-p2a-	This work

	mCherry$_{mFT}$-pA$_{bGH}$). pAB051 was then restricted using *MluI* and *HindIII* and inserted in the corresponding site of pTS2341.<br><br>(ITR-P$_{AcoO}$-P$_{CMVmin}$-SEAP-p2a-mCherry$_{mFT}$-pA$_{bGH}$-P$_{RPBSA}$-PuroR-pA$_{p9}$-ITR)	
pAB804	Stable Sleeping Beauty integration plasmid bearing AcoR and the red fluorescent marker protein mRuby fused via p2a to a puromycin resistance gene.<br><br>pAB003 was then restricted using *MluI* and *HindIII* and inserted in the corresponding site of pTS2339.<br><br>(ITR-P$_{mPGK}$-VP16$_{f\text{-}type}$-AcoR-pA$_{bGH}$-P$_{RPBSA}$-mRuby2-p2a-PuroR-pA$_{p9}$-ITR)	This work
pBS894	Sleeping Beauty-specific transposon encoding O$_{AcoR}$-P$_{hCMVmin}$ driven SEAP and mINS expression as well as constitutive P$_{RPBSA}$- driven YPet and PuroR expression.<br><br>O$_{AcoR}$-P$_{hCMVmin}$ was restricted using *MluI* and *EcoRI* from pAB101 and inserted into the corresponding sites of pBS828.<br><br>(ITR- O$_{AcoR}$-P$_{hCMVmin}$-SEAP-p2a-mINS-pA$_{bGH}$-P$_{PRPBSA}$-EGFP-p2a-ZeoR-pA$_{bGH}$-ITR)	This work
pBS1020	AcoR-inducible mammalian SEAP reporter protein expression vector from the P$_{YB\text{-}tata}$ minimal promoter.<br><br>O$_{AcoR}$ was obtained through annealing and phosphorylation of oAB025 (cgcgtggtaccctcgagactcttcatacgttgGAGACAAAATGAGACATATGTCTCATTTTGTCTCa) and oAB026 (tcgatGAGACAAAATGAGACATATGTCTCATTTTGTCTCcaacgtatgaagagtctcgagggtacca) and ligation into *MluI XhoI* restricted pBS237.<br><br>(O$_{AcoR}$-P$_{YB\text{-}tata}$-SEAP-pA$_{bGH}$)	This work

pBS1021	AcoR-inducible mammalian SEAP reporter protein expression vector from the $P_{tata\text{-}T7}$ minimal promoter.<br><br>O_{AcoR} was obtained through annealing and phosphorylation of oAB025 (cgcgtggtaccctcgagactcttcatacgttgGAGACAAAATGAGACATATGTCTCATTTTGTCTCa) and oAB026 (tcgatGAGACAAAATGAGACATATGTCTCATTTTGTCTCcaacgtatgaagagtctcgagggtacca) and ligation into *MluI XhoI* restricted pAna268.<br><br>(O_{AcoR}-$P_{tata\text{-}T7}$-SEAP-pA_{bGH})	This work
pBS1023	AcoR-inducible mammalian SEAP reporter protein expression vector from the Pgl4.23 minimal promoter.<br><br>O_{AcoR} was obtained through annealing and phosphorylation of oAB025 (cgcgtggtaccctcgagactcttcatacgttgGAGACAAAATGAGACATATGTCTCATTTTGTCTCa) and oAB026 (tcgatGAGACAAAATGAGACATATGTCTCATTTTGTCTCcaacgtatgaagagtctcgagggtacca) and ligation into *MluI XhoI* restricted pSP16.<br><br>(O_{AcoR}-$P_{gl4.23}$-SEAP-pA_{bGH})	This work
pBS1024	AcoR-inducible mammalian SEAP reporter protein expression vector with the P_{min} minimal promoter.<br><br>O_{AcoR} was obtained through annealing and phosphorylation of oAB025 (cgcgtggtaccctcgagactcttcatacgttgGAGACAAAATGAGACATATGTCTCATTTTGTCTCa) and oAB026 (tcgatGAGACAAAATGAGACATATGTCTCATTTTGTCTCcaacgtatgaagagtctcgagggtacca) and ligation into *MluI XhoI* restricted pVH254.<br><br>(O_{AcoR}-P_{min}-SEAP-pA_{bGH})	This work
pBS1025	AcoR-inducible mammalian SEAP reporter protein expression vector with 11 bp promoter spacer.	This work

	O_{AcoR} with an 11 base pair spacer was obtained through annealing and phosphorylation of oBS121 (CGCGTCTCGAGGGAGACAAAATGAGACATATGTCTCATTTTGTCTCATTCATACGTTA) and OBS122 (TCGATAACGTATGAATGAGACAAAATGAGACATATGTCTCATTTTGTCTCCCTCGAGA) and annealing into *MluI* and *XhoI* restricted $P_{hCMVmin}$-SEAP.<br><br>(O_{AcoR}-11bpSpacer-$P_{hCMVmin}$-SEAP-pA_{bGH})	
pBS1026	AcoR-inducible mammalian SEAP reporter protein expression vector with 22 bp promoter spacer.<br><br>O_{AcoR} with a 22 base pair spacer was obtained through annealing and phosphorylation of oBS123 (CGCGTCTCGAGGGAGACAAAATGAGACATATGTCTCATTTTGTCTCAATAGTAAACTCTTCATACGTTA) and oBS124 (TCGATAACGTATGAAGAGTTTACTATTGAGACAAAATGAGACATATGTCTCATTTTGTCTCCCTCGAGA) and annealing into *MluI* and *XhoI* restricted $P_{hCMVmin}$-SEAP.<br><br>(O_{AcoR}-22bpSpacer-$P_{hCMVmin}$-SEAP-pA_{bGH})	This work
pBS1027	AcoR-inducible mammalian SEAP reporter protein expression vector with 33 bp promoter spacer.<br><br>O_{AcoR} with a 33 base pair spacer was obtained through annealing and phosphorylation of oBS125 (CGCGTCTCGAGGGAGACAAAATGAGACATATGTCTCATTTTGTCTCACTCAATGATTCACAGTAAACTCTTCATACGTTA) and oBS126 (TCGATAACGTATGAAGAGTTTACTGTGAATCATTGAGTGAGACAAAATGAGACATATGTCTCATTTTGTCTCCCTCGAGA) and annealing into *MluI* and *XhoI* restricted $P_{hCMVmin}$-SEAP.<br><br>(O_{AcoR}-33bpSpacer-$P_{hCMVmin}$-SEAP-pA_{bGH})	This work

Abbreviations and additional information:

AcoR, acetoin catabolism regulatory protein; **VPR**, fusion of the three transactivator domains VP64-p65-Rta; **VP16**, herpes simplex virus type 1 derived transcriptional activator; **VP16$_{f\text{-}type}$**, minimal acidic activation domain of VP16; **CRE,** cAMP response element; **mCherry**$_{mFT}$, fluorescent

timer protein derived from mCherry; **MCS**, multiple cloning site; **mRuby2**, mRuby-derived red fluorescent protein; **Nluc**, luciferase from *Oplophorus gracilirostris* (NanoLuc); **p2A**, 2A self-cleaving peptide; **pA_{bGH}**, bovine growth hormone polyadenylation signal; **pA_{p9}**, synthetic polyadenylation signal; **P_{CMVmin}**, hCMV minimal promoter; **$P_{GL4.23}$**, pGL4.23 minimal promoter; **P_{min}**, pGL4.23-derived minimal promoter; **$P_{YB\text{-}tata}$**, synthetic minimal promotor; **$P_{tata\text{-}T7}$**, T7-derived minimal promoter; **$P_{hEF1\alpha}$**, human elongation factor 1 alpha promoter; **P_{hCMV}**, human cytomegalovirus immediate-early promoter; **P_{mPGK1}**, mouse phosphoglycerate kinase 1 promoter; **P_{SV40}**, simian virus 40 promoter; **PuroR**, puromycin resistance gene; **SEAP**, human placental secreted alkaline phosphatase; **VanA**, vanillic acid-controlled transcriptional activation; **ITR**, inverted terminal repeats; **mINS**, insulin variant optimized for production in HEK293T cells; **EGFP**, enhanced green fluorescent protein; **ZeoR**, zeocin resistance gene; **BlastR**, blasticidin resistance gene.

Oligomers: Capital letters indicate regions of interest. Underlined lower-case letters indicate nucleotides of the AcoR binding site replaced by non-interacting nucleotides. Lower-case letters indicate spacer elements including restriction sites and overhangs.

Supplementary References

Ausländer, S., Fuchs, D., Hürlemann, S., Ausländer, D., & Fussenegger, M. (2016). Engineering a ribozyme cleavage-induced split fluorescent aptamer complementation assay. *Nucleic Acids Research*, *44*(10), e94-e94. https://doi.org/10.1093/nar/gkw117

Chassin, H., Müller, M., Tigges, M., Scheller, L., Lang, M., & Fussenegger, M. (2019). A modular degron library for synthetic circuits in mammalian cells. *Nature Communications*, *10*(1), 2013. https://doi.org/10.1038/s41467-019-09974-5

Edgar, R., Domrachev, M., & Lash, A. E. (2002). Gene Expression Omnibus: NCBI gene expression and hybridization array data repository. *Nucleic Acids Res*, *30*(1), 207-210. https://doi.org/10.1093/nar/30.1.207

Haellman, V., Saxena, P., Jiang, Y., & Fussenegger, M. (2021). Rational design and optimization of synthetic gene switches for controlling cell-fate decisions in pluripotent stem cells. *Metabolic Engineering*, *65*, 99-110. https://doi.org/https://doi.org/10.1016/j.ymben.2021.03.009

Haellman, V., Strittmatter, T., Bertschi, A., Stücheli, P., & Fussenegger, M. (2021). A versatile plasmid architecture for mammalian synthetic biology (VAMSyB). *Metabolic Engineering*, *66*, 41-50. https://doi.org/https://doi.org/10.1016/j.ymben.2021.04.003

Saxena, P., Charpin-El Hamri, G., Folcher, M., Zulewski, H., & Fussenegger, M. (2016). Synthetic gene network restoring endogenous pituitary–thyroid feedback control in experimental Graves' disease. *Proceedings of the National Academy of Sciences*, *113*(5), 1244-1249. https://doi.org/10.1073/pnas.1514383113

Stefanov, B. A., Teixeira, A. P., Mansouri, M., Bertschi, A., Krawczyk, K., Hamri, G. C. E., Xue, S., & Fussenegger, M. (2021). Genetically Encoded Protein Thermometer Enables Precise Electrothermal Control of Transgene Expression. *Advanced Science*, 2101813. https://doi.org/10.1002/advs.202101813

Subach, F. V., Subach, O. M., Gundorov, I. S., Morozova, K. S., Piatkevich, K. D., Cuervo, A. M., & Verkhusha, V. V. (2009). Monomeric fluorescent timers that change color from blue to red report on cellular trafficking. *Nature Chemical Biology*, *5*(2), 118-126. https://doi.org/10.1038/nchembio.138
